# Case-control study on uveal melanoma (RIFA): rational and design

**DOI:** 10.1186/1471-2415-4-11

**Published:** 2004-08-19

**Authors:** Andrea Schmidt-Pokrzywniak, Karl-Heinz Jöckel, Norbert Bornfeld, Andreas Stang

**Affiliations:** 1 Institute of Medical Informatics, Biometry and Epidemiology, University Hospital, University of Duisburg-Essen, Hufelandstr. 55, 45122 Essen, Germany; 2 Division of Ophthalmology, University Hospital, University of Duisburg-Essen, Hufelandstr. 55, 45122 Essen, Germany; 3 Institute of Medical Epidemiology, Biometry and Informatics, Medical Faculty, Martin-Luther-University Halle-Wittenberg, Magdeburgerstr. 27, 06097 Halle, Germany

## Abstract

**Background:**

Although a rare disease, uveal melanoma is the most common primary intraocular malignancy in adults, with an incidence rate of up to 1.0 per 100,000 persons per year in Europe. Only a few consistent risk factors have been identified for this disease. We present the study design of an ongoing incident case-control study on uveal melanoma (acronym: RIFA study) that focuses on radiofrequency radiation as transmitted by radio sets and wireless telephones, occupational risk factors, phenotypical characteristics, and UV radiation.

**Methods/Design:**

We conduct a case-control study to identify the role of different exposures in the development of uveal melanoma. The cases of uveal melanoma were identified at the Division of Ophthalmology, University of Essen, a referral centre for tumours of the eye. We recruit three control groups: population controls, controls sampled from those ophthalmologists who referred cases to the Division of Ophthalmology, University of Duisburg-Essen, and sibling controls. For each case the controls are matched on sex and age (five year groups), except for sibling controls. The data are collected from the study participants by short self-administered questionnaire and by telephone interview. During and at the end of the field phase, the data are quality-checked.

To estimate the effect of exposures on uveal melanoma risk, we will use conditional logistic regression that accounts for the matching factors and allows to control for potential confounding.

## Background

Although a rare disease, uveal melanoma of the eye is the most common primary intraocular malignancy in adults, with an incidence rate of up to 1.0 per 100,000 person years (age-standardized, world standard) in Europe [1]. Only a few consistent risk factors have been identified for this disease. One set of uncommon risk factors include predisposing diseases like the dysplastic nevus syndrome, atypical ocular nevi, ocular and oculardermal melanocytosis [2,3]. Another set of host risk factors are ancestry, light skin and iris pigmentation [4-6]. In addition, a number of environmental factors including UV radiation [7,8] are weakly or inconsistently associated with uveal melanoma.

Some uveal melanoma are associated with neurofibromatosis. However, the vast majority of familial cases reported are non-syndromic [9]. Some recent studies suggests that mutations in the breast cancer susceptibility locus, BRCA2 on chromosome 13, may be involved in the development of uveal melanoma [9,10].

Occupation may be also relevant, and may include chemical work [11,12], arc welding [8,12] and agriculture and farming work [13,14]. Two recent studies found an increased risk of uveal melanoma among cooks [8,15]. Electromagnetic waves with frequencies of 300 kilohertz (kHz) to 300 gigahertz (GHz) are called radio-frequency radiation. Typical occupational sources transmitting radio-frequency radiation in Germany include walkie-talkies in the military and security services, in plants, radio sets on ships, transporters, freight trains, police cars and wireless phones including cellular phones (C-net: 450–465 MHz, since the 1990 ies D-net: 890–960 MHz and E-net: 1710–1800 MHz) and cordless phones (800–1900 MHz) with different modulation types.

The population-wide introduction of analog and digital mobile phone techniques in the recent years, which has been coined as the mobile revolution [16], has resulted in an increasing number of people who fear that radio-frequency radiation (RFR) may have adverse health effects [17]. There is currently much uncertainty about the role, if any, of radio frequency transmitted by radio sets or mobile phones in human carcinogenesis. The assessment of the potential association of radio-frequency radiation and cancer risk is hampered by uncertainties about effective electromagnetic frequency ranges, the lack of a clear biological mechanism, as well as by difficulties of exposure assessment.

Until now, the majority of epidemiological cancer studies focussed on brain cancer because the brain may be exposed to RFR [18-20]. With the exception of one study by Hardell et al. [21], all brain cancer studies showed no association between RFR as emitted by mobile phones and brain tumour risk until now. In contrast, the pooled analysis of two recent German case-control studies on the aetiology of uveal melanoma showed that frequent use of radiofrequency radiation devices including radio sets and mobile phones at the work place is associated with an about 4.2-fold elevated risk for uveal melanoma [22]. However, several methodological limitations including a small study size and a crude exposure assessment complicated the interpretation of these findings. Here we present the study design of an ongoing incident case-control study on uveal melanoma (acronym: RIFA study) that focuses on radiofrequency radiation as transmitted by radio sets and wireless telephones. We expect to publish the results of the study in summer 2005.

## Methods/Design

### Study questions

The RIFA study is planned to answer several etiologic questions with a special focus on electromagnetic radiation especially radio-frequency radiation as emitted by mobile phones and radio sets. First, is the finding of an increased risk of uveal melanoma among subjects with frequent use of RFR devices reproducible? Second, if there is an association, is this association site-specific in terms of laterality of the uveal melanoma and major site of mobile phone use? Third, if there is an association, is there a dose-response relationship between RFR and uveal melanoma risk? Another set of etiologic question relates to pigmentation characteristics including iris colour, hair colour, tendency of the skin to burn and to tan, freckling, number of cutaneous nevi. A further study questions relates to exposure to work and leisure time related ambient ultraviolet radiation and uveal melanoma risk. In addition, our study focuses on several occupational exposures or jobs that are suspected to be associated with an increased risk of uveal melanoma [23,15] including working in the chemical industry, farming, coal mining, welding, cooking, working in the health service sector etc. Finally, we focus on the association between cancer history of the index persons and their relatives (especially breast cancer) and risk of uveal melanoma.

### Case recruitment

The case recruitment is hospital-based and takes place in the Division of Ophthalmology, University of Essen which is a the referral centre for eye cancer in Germany, currently treating about 400–500 eye cancer patients per year. Eligible uveal melanoma cases have to fulfil several criteria. Patients with newly diagnosed first uveal melanoma located in the choroid, iris, and/or ciliary body [24] during the recruitment period from September 25^th^, 2002 to September 24^th^, 2004 are eligible, if they are referred to the Division of Ophthalmology, University of Duisburg-Essen during the recruitment period, are aged 20–74 years at diagnosis, are living in Germany, and are capable to complete the interview in German. The majority (about 70–80%) of uveal melanoma treated at the University Hospital Essen receive episcleral plaque therapy without histological verification. For this reason we did not include a reference pathologist who reviews the diagnostic certainty of the cases. Experiences from our previous case-control studies showed that there is a nearly perfect agreement between the local eye doctors in the reference centre and the international reference pathologist (Dr. Ian Cree, London) [22].

We considered a diagnosis of uveal melanoma as definite if the results of the clinical examination of the eye (ophthalmoscopy) and ultrasound (sometimes supplemented by fluorescence angiography, computertomography or magnetic resonance imaging) were unambiguous. The inclusion and exclusion criteria of the cases are listed in table 1.

### Control recruitment

**Figure 1:**
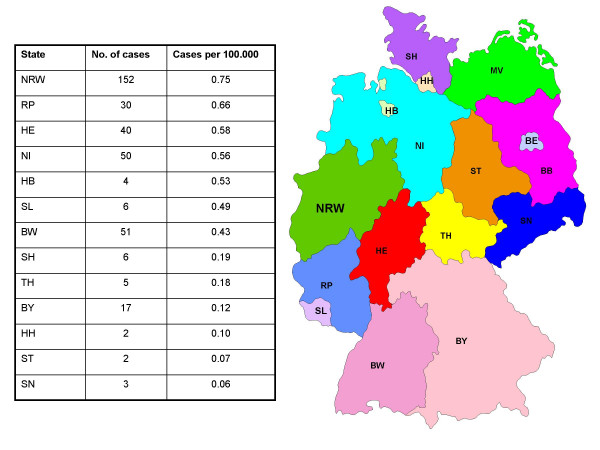
Geographic distribution of 368 uveal melanoma cases referred to the Division of Ophthalmology, University of Duisburg-Essen, September 25^th^, 2002 through March 31, 2004. Federal states, not listed in this table provided no cases. NRW = North Rhine-Westphalia, RP = Rhineland-Palatinate, HE = Hessen, NI = Lower Saxony, HB = Bremen, SL = Saarland, BW = Baden Wuerttemberg, SH = Schleswig-Holstein, TH = Turingia, BY = Bavaria, HH = Hamburg, ST = Saxony Anhalt, SN = Saxony

Interim analyses showed that the majority of cases comes from the territory of former West Germany. Figure 1 displays the geographic distribution of cases treated for uveal melanoma at the Division of Ophthalmology, University of Duisburg-Essen, from September 24^th^, 2002 through March 31^th^, 2004. Assuming comparable incidences of uveal melanoma in the federal states of Germany, the crude rate (referred cases divided by population at risk aged 20–74 years) may be considered as an indicator of the referral effect.

Obviously, the referral effect varies by federal states. However, it is difficult to judge whether the case referral to the Division of Ophthalmology, University of Duisburg-Essen is a random sample of all newly diagnosed uveal melanoma cases in Germany. We therefore decided to recruit three different control groups. First, if we assume that cases treated in Essen are a random sample of all cases in Germany, a population-based control group would be the most appropriate control group. For this approach, we randomly select controls from mandatory lists of residence that cover the total population of the city or local district. These lists are regarded as the most complete sampling frame for population-based studies in Germany.

Second, if the referred cases are not a random sample of all newly diagnosed cases of uveal melanoma in Germany, a control group sampled from those ophthalmologists who referred cases to the Division of Ophthalmology, University of Duisburg-Essen, would be most appropriate [25,26]. To increase the statistical power, we decided to include two controls per case.

In addition, we recruit sibling controls of cases (matching ratio 1:1) in order to assess whether genetic factors may confound the effect of exposure. The sibling controls are matched in genetic background. The inclusion criteria of the three control groups are presented in table 2.

### Power calculations

Based on our former uveal melanoma case-control studies [15,22] we estimated to identify 480 eligible cases within a recruitment period of 24 month. With an anticipated response proportion of about 80%, we expect to interview 380 cases overall.

Population-based prevalence estimates of mobile phone use in the general population are scant. A recent telephone survey from 2001 showed that 82% of male and 74% of female participants aged 14–44 years use mobile phones; within the age group 45–59 years, 63% of male and 58% of female participants use mobile phones. The oldest age group (> = 60 years) shows considerable lower prevalences of mobile phone use (men: 49%, women: 25%) [27]. To determine the statistical power of the case-control study, we assumed several mobile phone prevalence estimates in the control group. We conducted all power calculations two-sided according to formulas of Woodward [28]. We chose α to be 5% and 1-β to be 90%. Detectable increases of odds ratio estimates by varying prevalences of mobile phone use in the control group are presented in table 3. A case-control interview ratio of 380 to 760 would enable us to detect increased odds ratios in the range of 1.5 to 2.2 depending on the exposure prevalence in the control group (table 3).

### Exposure assessment

Table 4 presents a list of exposures that are assessed in the RIFA study.

The questionnaire on mobile phone use is the same instrument which has been used by the international case-control study on brain cancer and mobile phone use sponsored by the International Agency for Research on Cancer, called Interphone study [29]. In contrast to the Interphone study, we do not perform personal interviews but telephone interviews. For this reason, we cannot show photographs of different types of mobile phones in order to assess the detailed type of mobile phone used.

Compared to other studies on the aetiology of uveal melanoma, the RIFA study uses a detailed assessment of pigmentary characteristics. The self-administered questionnaire contains a eye and hair colour card that allows the participants to choose the most appropriate colour of their eyes and hairs at age 20 years. During the telephone interview, the skin reaction to sun exposure (tanning ability, burning tendency) is asked according the concept of Fitzpatrick [30]. Fitzpatrick's original question contains both items (tanning ability, burning tendency) within a single question which is of methodological concern because the categorical answers given by the Fitzpatrick question may not presents all types of skin reactions as has been demonstrated by Rampen et al. [31]. To reduce this potential misclassification, we separated the Fitzpatrick items into two questions that separately ask about burning tendency and tanning ability.

For the assessment of nevi of the upper arms and the dorsum of the feet with a diameter of at least 3 mm, participants receive a template with a 3 mm hole that enables them to count all nevi of this minimum size. In addition, the CATÍ (computer assisted telephone interview) includes a detailed history of sunburns in the recent 15 years before interview and tendency to freckle as a child. The CATI contains a question on eye colour with the typical categorical answers as has been used by several others (blue, grey, green, hazel, brown, black, [22] that will enable us to study the agreement between eye colour assessment by colour cards and colour categories.

**Figure 2:**
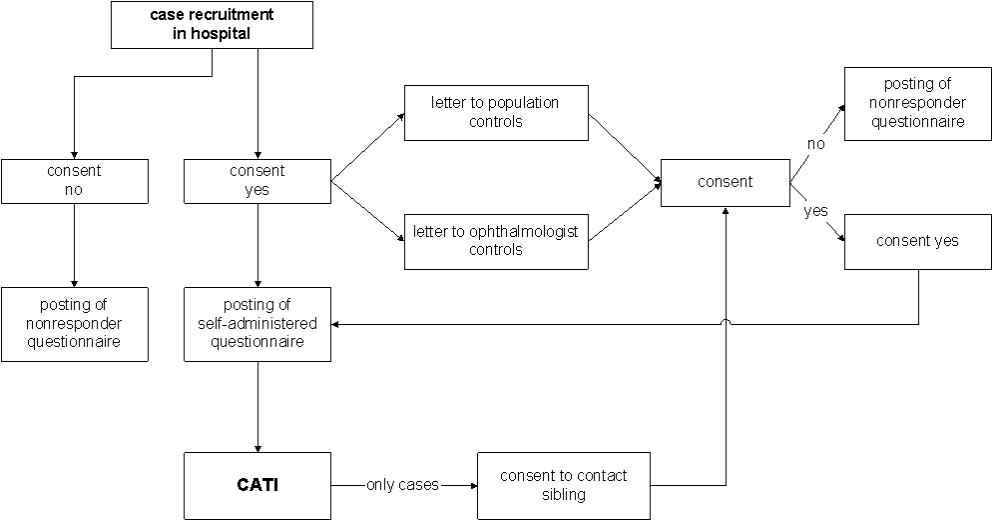
Course of exposure assessment of the RIFA case-control study

The course of the exposure assessment is displayed in figure 2. The exposure assessment starts with a self-administered questionnaire among subjects who agreed to participate and gave written informed consent. Subjects are chronologically asked about each job held for at least six months and included questions on the job tasks and industries as has been done in previous studies on the aetiology of uveal melanoma [22]. In addition, subjects are asked questions related to eye colour, hair colour, ever use of mobile phone, wireless telephone and radio set, and number of nevi. Subjects who have sent back the self-administered questionnaire undergo a computer-assisted telephone interview which takes about 30–40 minutes.

For subjects who reported selected work tasks (e.g. cooking and food processing, welding and others), we use 16 job-specific supplementary questionnaires to obtain details of the job tasks and materials used.

### Quality assurance

The quality control program includes several procedures. The study is designed to fulfill the recommendations of the German Good Epidemiologic Practises [32].

Eight month before the main study started, a manual of standard operating procedures (MOP) was written and a pilot study of four weeks was conducted to test the field work and exposure questionnaires. After some minor revisions of the MOP, report forms, and questionnaire instruments, the principal investigator and participating epidemiologists had to sign that they fully agree with the final version of the MOP.

Interviewers of the study were introduced into the field work and were blinded against our study hypotheses. After an initial interviewer training course, interviewers are regularly monitored and receive regular training courses.

The recruitment progress, given as number of registered cases and controls, distribution of inclusion and exclusion criteria, response proportions, is monitored monthly. The analysis of nonresponse reasons is supplemented by an short questionnaire for subjects not willing to participate. This questionnaire includes few demographic and exposure items that help us to assess potential selection effects due to nonresponse.

A plausibility control of the interview data is done quarterly and is the basis for the regular training courses of the interviewers.

The completeness of case registration is checked by regular comparison of the list of registered cases with lists of admissions to the referral centre. In addition we compare our list of cases with data of the hospital information system that includes information on diagnoses.

The self-administered short questionnaires are visually edited by the study personnel before the telephone interview starts. The visual editing includes a completeness check and coding of the life-long job history. For each job period, the occupation and branch of industry is coded according to ISCO-68 [33] and NACE 1993 [34]. These classifications have been repeatedly used in occupational case-control studies. Self-administered questionnaires with incomplete information or missing data are marked and questions are prepared for the telephone interviewer who is responsible to ask these questions before the main telephone interview starts.

The CATI contains internal quality checks that prevent data entry errors. For example, interviewers are not able to fill in the detailed questions on mobile phones, if the entry question on ever having used mobile phone has been answered with no.

### Planned analyses

At the end of the field phase, the data are quality-checked. To estimate the effect of exposures on uveal melanoma risk, we will use conditional logistic regression that accounts for the matching factors and allows to control for potential confounding. We will classify people exposed to an occupational category if they ever worked within this category for at least six months. The quantification of mobile phone use will be based on average number of phone calls and average duration of phone calls per time unit. The association between pigmentary characteristics and uveal melanoma risk will be assessed by detailed matrix containing information on hair colour at age 20 years, eye colour, freckling tendency, and skin colour. Final results of these analyses are scheduled to be published in summer 2005.

## Competing interests

None declared.

## Pre-publication history

The pre-publication history for this paper can be accessed here:



## Figures and Tables

**Table 1:** Inclusion criteria of the cases

**Characteristics**	
Diagnosis	Posterior and anterior uveal melanoma ^1)^
	ICD10
	C69.3 (Choroid)
	C69.4 (Ciliary body)
	ICD-O-3: Localisation
	C69.42 (Iris)
	C69.43 (Ciliary body +/- further choroid sections)
	C69.3 (Choroid)
	ICD-O-3 Histological types
	8720/3 (Malignant melamoma, NOS)
	8770/3 (Mixed epithelioid and spindle cell melanoma)
	8771/3 (Epithelioid cell melanoma)
	8773/3 (Spindle cell melanoma, type A)
	8774/3 (Spindle cell melanoma, type B)
Date of the first diagnosis	25.09.2002 – 24.09.2004 (24 months)
Age at diagnosis	20–74 years^2)^
Sex	Man or woman
Residence	Germany
Language	Being capable to complete the interview in German

1) Also patients with uveal melanoma who were never referred to Division of Ophthalmology, University of Duisburg-Essen, for diagnostics. 2) Reference date for calculation of age in cases in the first visit at Division of Ophthalmology, University of Duisburg-Essen due to uveal melanoma, as long as there is no clue that the uveal melanoma was diagnosed more than three month earlier.

**Table 2:** Inclusion and exclusion criteria of three different control groups of the RIFA case-control study

**Characteristics**			
	**Population control**	**Ophthalmologist control**	**Sibling control**

Age	Within 5 year age group of case^1^	Within 5 year age group of case^1^	Age within +/- ten years compared to age of case. If more then one sibling lies in this range, the one closest to the case is chosen, unless he/she refuses. If so, the sibling fitting second best in age is chosen.
Sex	The same sex as case	The same sex as case	The same sex as the case; when a same sex sibling does not exist or refused, then the opposite – sex sibling is chosen.
Reference period		Visit at the ophthalmologist's within 10 working days (possibly more)^2 ^before reference date set by case diagnosis.	
Diagnosis	No history of cancer	No history of cancer	No history of cancer
Language	Being capable to complete the interview in German	Being capable to complete the interview in German	Being capable to complete the interview in German
Region of residence	Residence matched to case^3^	Germany	

1) The reference date is the date of the case diagnosis 2) With small ophthalmologist's practices it may become necessary to increase the span of ten working days in order to recruit ten eligible patients. 3) As residence matching is not performed at the level of single cities, controls will be taken from a sample in a city comparable size within a radius of 60 km around the case's habitation, if such a sample exists.

**Table 3:** Detectable increased Odds Ratios in relation to expected study size and expected prevalences of mobile phone use

Mobile phone prevalence in the control group	Detectable Odds Ratio (Cases : Controls)			
	480:960	380 : 760	300 : 600	250 : 500

0.05	2.0	2.2	2.4	2.6
0.10	1.7	1.8	2.0	2.1
0.15	1.6	1.7	1.8	1.9
0.20	1.5	1.6	1.7	1.8
0.25	1.5	1.6	1.7	1.7
0.30	1.5	1.5	1.6	1.7
0.35	1.5	1.5	1.6	1.7

Calculations according to Woodward; α = 0.05, β = 0.10; two-sided

**Table 4:** Overview of exposure assessment, RIFA case-control study

	Measurement of exposure
Phenotypical characteristics	Hair colour with 20 year, eye colour, skin colour
	Ability to tan, propensity to burn
	Number of nevi > 3 mm on the upper arms & dorsum of the feet
	Freckling as a child
Sun exposure	Occupational outside work, holidays in the last 15 years
	Sunburns in the last 15 years
Artificial UV radiation	Exposure sources to artificial UV radiation as welding & tanning devices
Use of protective devices	Use of sunglasses or hats
Eye burns	Snow blindness, welding burns or sunburns of the eyes
Radio-frequency radiation	Use of mobile phones, wireless telephones
Jobs-specific supplementary	
Main task	Cooking, welding, radar, working with electronic equipment, working with microwave
Job groups	Mining, farming, working with farm animals, agriculture, chemical industry, health care
Medical history	Index person and family cancer histories
Social class	Highest school degree, highest professional degree, income
